# Weight Stigma in Pregnancy: A Narrative Review of Consequences, Drivers, and Potential Solutions

**DOI:** 10.1007/s13679-026-00772-x

**Published:** 2026-10-26

**Authors:** Briony Hill, Haimanot Hailu, Bec Jenkinson, Ahlia Griffiths, Emma Davidsen, Jodie Dodd, Megan Mitchell, Leonie Callaway, Lauren Kearney, Helen Skouteris

**Affiliations:** 1 Health and Social Care Unit, School of Public Health and Preventive Medicine, Monash University, Melbourne, Australia; 2 Faculty of Health, Medicine and Behavioural Sciences, The University of Queensland, Brisbane, Australia; 3 Prevention, Health Promotion & Community Care, Steno Diabetes Center Copenhagen, Herlev, Denmark; 4 Women’s and Babies Division, Women’s and Children’s Hospital, Adelaide, Australia; 5 Robinson Research Institute, College of Health, Adelaide University, Adelaide, Australia; 6 Department of Women’s and Newborn Services, Royal Brisbane & Women’s Hospital, Herston, Australia; 7 School of Nursing, Midwifery and Social Work, The University of Queensland, Brisbane, Australia; 8 Warwick Business School, The University of Warwick, Coventry, UK

**Keywords:** Weight prejudice, Weight stigma, Weight-inclusive, Gestational weight gain, Pregnancy

## Abstract

**Purpose of Review:**

This narrative review summarises current knowledge of weight stigma in pregnancy including consequences, drivers, and potential solutions.

**Recent Findings:**

Weight stigma in pregnancy has negative physical, psychosocial, and behavioural sequelae for mothers and their infants. It is an equity issue that compounds disadvantage experienced by marginalised communities. A focus on gestational weight gain in maternity care, particularly for women with a high body mass index has created an environment that perpetuates implicit and explicit weight stigma, negatively impacting women’s care. The community, family members, and media are also key weight stigma perpetrators. This stigma pervades societal norms, influencing healthcare environments, practice, and policy.

**Summary:**

Tackling weight stigma in maternity care requires a shift from weight-centric, stigmatising approaches to more weight-inclusive, woman-centred, and holistic health-focused care. This should be supported by approaches addressing societal and cultural norms and policy change, ultimately leading to improvements in the health and wellbeing of women during pregnancy and beyond.

## Introduction

Weight stigma is a health, social, and equity issue. It is characterised by negative weight-related attitudes and beliefs manifested as prejudice, rejection, and negative stereotypes towards individuals with a larger body size [1]. Weight stigma is usually, but not always, discussed in relation to individuals described with a body mass index (BMI) over 25 kg/m^2^ or living with overweight or obesity. Despite diverse language preferences, using the terms larger-bodied or higher weight is generally accepted by people with lived experience as more appropriate and less stigmatising than terms such as “obese” [2]. Drawing on this literature and our ongoing relationships with women with lived experience, the term “larger-bodied” will be used in this paper [3]. The term obesity, and/or higher BMI, will be used only when referring to research that describes weight in relation to BMI ≥ 30 kg/m^2^. Weight stigma is experienced by women across the BMI spectrum, although women with higher BMI tend to experience a greater number of sources and frequency of weight stigmatising experiences [4].

Weight stigma is particularly salient for pregnant women^1^ due to prevailing social norms and expectations about weight and the close monitoring of pregnancy weight gain in healthcare settings [5]. These include the public perception that women who care about the health of their baby should not gain too much weight in pregnancy, should “bounce back” to a thin body size soon after giving birth, and that being slim equates to being a good mother [5, 6]. Although body size is largely outside of individual control and likely explained by many other factors including genetics [7], perceptions otherwise drive a narrative that having a body size outside of societal prescriptive norms is unhealthy, usually because a woman has not “tried hard enough” to be slim [8]. This narrative reinforces the notion that blaming or shaming women will motivate them to lose weight and/or limit their gestational weight gain (GWG), thereby improving birth outcomes [9, 10]. However, evidence shows that weight stigma does not motivate weight loss and instead might cause physiological, psychological, and behavioural harm [11]. It also contributes to healthcare inequities that disproportionately affect larger-bodied women [12].

While weight stigma in pregnancy has important health implications and is common in maternity care [2, 13], it is fundamentally a social issue [14]. Weight stigma manifests through culture, communication, cues, and societal discourse [8]. Weight stigma exists across all sociocultural layers, including in the policies that guide the way we live, work, and practice and receive healthcare; our communities; at work or school; with our families and peers; and internalised among individuals [5]. Societal norms about weight and body size in pregnancy are the root of a much larger issue of stigmatising evidence, discourse, and healthcare impacting pregnant women [15, 16]. Hence, addressing weight stigma in pregnancy is an essential component of optimising maternal wellbeing. The purpose of this review is to provide a summary of current knowledge of weight stigma in pregnancy including its harmful consequences, drivers, and potential solutions. We conducted a narrative expert review drawing upon literature published within the last five years and older landmark literature where suitable. Level one evidence was prioritised over single primary studies where possible, with the most recent evidence discussed.

## The Lived Experience and Consequences of Weight Stigma for Pregnant Women

### Lived Experience Voice

The voices of women with lived experience of weight stigma in pregnancy provide essential understanding of its drivers, consequences, and potential solutions [17]. Research incorporating lived experience, either through participatory research methods [17, 18] or engagement with consumers across the research cycle, can provide rich, strengths-based, and empathy driving data, existing as a unique form of evidence to inform future practice and policy change [19]. A 2023 peer-reviewed paper written by consumers for researchers highlighted the need to listen to larger-bodied women as an approach to addressing weight stigma in maternity care [3].

For larger-bodied women, current approaches in maternity care are experienced as weight-centric and stigmatising. Women commonly report encountering weight stigma in a variety of contexts [20] and in maternity care it is enacted through policy [15] and woven into interpersonal interactions and environments [17]. Women describe feeling reduced to their BMI or weight [3], which obscures an individualised assessment of their health and inadvertently minimises their broader health histories and priorities [17]. Further, BMI-based risk stratification diminishes women’s involvement in decision making about their care [21] and excludes them from evidence-based models of care and desired pregnancy experiences such as midwifery-led care and water immersion for labour and birth [22, 23].

Practical strategies to reduce stigma have been identified within lived experience narratives and research alike. These include ensuring healthcare environments are inclusive of diverse body sizes, with suitable equipment and furniture [18]. A sense of welcome and validation can also be created by including positive imagery of diverse body sizes in community-facing health information resources [18]. Training for maternity care providers should promote empathy and respectful communication, as well as challenging weight stigma attitudes and beliefs [2]. Importantly, women do not generally seek to avoid weight-related discussions when clinically indicated; rather, they call for dignified and sensitive conversations. Avoidance of these conversations is also a form of stigmatisation in maternity care [24].

### Consequences of Weight Stigma

While lived experience voices are essential to understanding weight stigma in pregnancy, academic and clinical experts also argue that the harms caused by weight stigma may exceed those caused by obesity [25], although there is a dearth of direct comparative evidence. Furthermore, the harms of weight stigma unequivocally exacerbate health consequences linked to obesity and the consequences of both are deeply intertwined [26, 27]. Weight stigma negatively impacts women’s wellbeing across biological and socioecological systems. At its core, weight stigma is thought to disrupt cortisol production and induce a physiological stress response [6], however, observational and experimental studies exploring the underlying mechanisms are not consistent [27–30]. The discordant findings may potentially be explained by failure to incorporate the intersectionality of weight stigma and include the multifaceted nature of marginalised identities [30].

Weight stigma also has substantial impacts on mental health, including depression, anxiety, and disordered eating [6]. These associations are found in both the general population and in pregnancy specifically [31, 32] and are possibly explained by greater perceived stress [33]. Incollingo Rodriguez and colleagues surveyed 501 pregnant and postpartum women in the US and found that, after adjusting for key clinical and demographic factors, exposure to weight stigma from more sources was associated with greater depressive symptoms, maladaptive and emotional eating, and increased perceived stress [31]. Weight stigma also impacts women’s ability to maintain optimal nutrition and physical activity behaviours that then further impedes their pregnancy health, compounded by the known link between poorer mental wellbeing and decreased participation in health behaviours [34, 35]. Women also report disengaging from healthcare, doctor-shopping, or delays in seeking maternity care due to weight stigma [5]. Beyond its impact on pregnant women, weight stigma has also been linked to adverse downstream birth and childhood outcomes [5, 26], potentially via activation of stress hormones and physiological pathways that link maternal exposures to infant health outcomes [26].

Despite accumulating evidence of the negative associations between experiencing weight stigma and poorer maternal psychosocial and behavioural outcomes [31, 32], weight stigma has rarely been explored as a direct predictor or mediator of the relationship between maternal BMI and adverse pregnancy outcomes. A Swiss cross-sectional survey of 1352 women found that increased frequency of weight stigmatisation during labour and birth significantly and partially mediated the relationship between pre-pregnancy BMI and caesarean birth [36]. Cross-sectional self-report data (*n* = 358 postpartum women in the US) has also demonstrated an association between weight stigma concerns in pregnancy and the odds of being diagnosed with gestational diabetes mellitus (GDM) [37]. However, this study found no association between weight stigma concerns and caesarean birth, pre-term birth, low birthweight, or fetal macrosomia. Future research using longitudinal designs and objective measures is urgently needed to explore these relationships further.

## Weight Stigma as an Issue of Equity

While weight stigma has consequences for maternal health, it is also an issue of equity [38]. Weight stigma is recognised as a social determinant of health, intersecting with and disproportionately impacting communities that are marginalised by sex/gender, race/ethnicity, social class, and disability [38–40]. It also affects determinants of health such as financial and social resources [38]. Given that marginalised communities are already at increased risk of poorer pregnancy and birth outcomes compared to their counterparts from non-deprived backgrounds [41, 42], unchecked weight stigma has the potential to further compound these inequities.

In pregnancy, one of the primary ways in which larger-bodied women are prevented from accessing equitable care is through service restrictions. While many BMI-related healthcare service restrictions are based on empirical evidence, many are not. Examples of body size/weight/BMI-related healthcare restrictions include denial of access to preferred models of care (e.g., continuity of midwifery care); being directed into high-risk care pathways (e.g., obstetrician-led care) based only on BMI; being directed to access care at a hospital geographically distant from home [43, 44]; and eligibility screening for epidurals [45] and water births [24]. Barriers to access may be explicit (e.g., via policies) or be a result of environmental barriers such as incorrectly sized equipment that may either prevent adequate care being provided and/or discourage future care seeking [46, 47]. Limits on BMI may also require women to spend additional time or financial resources seeking alternative care [48, 49]. Additionally, clinicians often do not engage in conversations around service access restrictions in a sensitive and caring manner [18], which further stigmatises women. There is compelling lived experience evidence which highlights the deeply rooted and traumatic impact of denying preferred care to pregnant women based purely on BMI, discounting the woman as whole and ignoring the absence of other risk factors [50].

## Drivers of Weight Stigma

### Pre-Pregnancy BMI

The underlying factors that fuel the stigmatisation process are the drivers of weight stigma [51]. Pre-pregnancy BMI in the overweight or obesity categories is associated with adverse pregnancy outcomes, including pre-eclampsia, GDM, and caesarean birth [52, 53]. Observational studies, which are the basis of this evidence, predominantly present risk using relative risk or odds ratio, over-estimating risk when compared to absolute risk, particularly at the points of transition between BMI categories [53–55]. The association between BMI and adverse pregnancy outcomes is also highlighted through research and clinical practice guidelines [56] and is accompanied by a narrative created by weight loss literature that often focusses on energy imbalance (where energy intake exceeds energy expenditure) and presents the predominant solution as weight management, diet, and exercise [5]. This narrative, also reinforced by public health messaging, dominates over the growing evidence that body weight and obesity are the result of a complex interplay between multiple (epi)genetic, metabolic, and environmental causes [7, 57]. Hence, the continued rhetoric that women are individually responsible for a high BMI despite it being largely beyond individual control reinforces biased perspectives that women are to blame for their adverse pregnancy outcomes and that they could avoid them by simply losing weight or limiting GWG. However, acknowledging the complex array of factors that determine an individual’s body size and the poor predictive power of BMI [58] can reframe weight-related discussions towards the shared goal of optimal outcomes for larger-bodied women and their babies.

### Gestational Weight Gain

Along with BMI, high GWG is widely regarded via observational evidence as a behaviourally-linked outcome that contributes to adverse pregnancy outcomes such as caesarean birth and higher infant birthweight [59]. Weight management is recognised as a component of health across the lifespan, and it follows that endeavours to similarly restrict weight gain, particularly for larger-bodied women, during pregnancy could be a successful approach for reducing adverse pregnancy outcomes. We present two high-quality studies published in 2024 that call this association into question. Firstly, a secondary data analysis of individual participant data from 20 randomised controlled trials (4370 women) used regression-based modelling to determine the extent to which the observed effect of maternal BMI on pregnancy outcomes was mediated via GWG [53]. The key findings of this paper indicated that women with higher BMI had lower rates of GWG, as has been reported elsewhere [60]. However, it is often overlooked that the occurrence of excessive GWG reflected the thresholds for transitions between BMI categories [53]. Furthermore, the analyses produced no evidence of a relationship between GWG and the occurrence of adverse pregnancy outcomes. These findings highlight that there is likely an absence of a causal relationship between GWG and pregnancy outcomes and therefore GWG is not an appropriate intervention target for prevention. While this message is not intended to subvert goals for optimising antenatal nutrition and physical activity, this research stresses a need to identify the causal mechanisms underpinning the relationship between BMI and adverse pregnancy outcomes, which is multifactorial, reflecting elements of genetic predisposition, psychosocial factors, and weight stigma, all of which require further detailed evaluation. Furthermore, the aetiology of weight gain in pregnancy should consider the significant interplay between physiological responses unique to pregnancy [61]. For instance, GWG is a composite outcome influenced by multiple factors, including the growing products of conception such as fetus, placenta, and amniotic fluid as well as physiological changes, all of which contribute significantly to overall GWG [62].

Clinical and research discourse has maintained that dietary and physical activity modification can improve pregnancy outcomes, a position reinforced by multiple systematic reviews demonstrating modest benefits across a range of maternal and perinatal indicators [63–65]. We present a second study that challenges the status quo for nutrition and physical activity (lifestyle) behavioural interventions in pregnancy for weight management [66]. With a focus on interrogating trial quality and methodological rigour—elements inherently critical when evaluating heterogeneous lifestyle interventions—the researchers sought to re‑evaluate the evidence. Incorporating only high quality, low risk of bias studies determined by fastidious investigation of data available for included studies, meta-analyses were conducted (for GWG) with 78 studies and incorporating data available from 17,775 randomised participants. For combined diet and physical activity behavioural interventions, the weighted mean difference in GWG was − 1.10 kg (95%CI -1.62 to -0.58) indicating a small and clinically insignificant intervention effect. Furthermore, there was no evidence of an intervention effect on the prevalence of GDM, pre-eclampsia, caesarean birth, or infant birth weight measures. These findings call into question, and at least provide reason to pause and critically consider, whether advising women with healthy pregnancies to manage their GWG via behavioural interventions is prudent. This blanket advice, in the absence of nuanced consideration of factors broader than weight alone, may unintentionally reinforce harmful and stigmatising narratives about weight control that ultimately perpetuate women’s perceived failure to protect their unborn baby. Ceasing regular weighing in pregnancy unless there are other clinical indicators would therefore reflect the findings reported above, supporting midwives’ perspectives of the (lack of) utility of regular weighing in pregnancy [67] and recognising its contribution to stress and embarrassment for some women, while also being a risk factor for triggering eating disorders and disordered eating [2]. Hence, careful clinical consideration needs to be given to regular weighing and it may indeed be appropriate in some circumstances, for example, in instances of hyperemesis gravidarum where monitoring maternal weight loss forms part of the assessment of disease severity and response to treatment [68].

### Socioecological Contributors to Weight Stigma

Pregnant women commonly experience weight stigma across all socioecological levels from the broader community, healthcare settings, workplaces, media, family, friends, peers, and their own or other pregnant mothers [4, 5, 20, 69]. These levels span intrapersonal, interpersonal, institutional and organisational, community, and public policy factors. A landmark US study found that 65% of pregnant and postpartum women experienced weight stigma most commonly from society in general (i.e., community members), followed by the media, healthcare, strangers, and immediate family [4].

Beyond healthcare settings, community members are a major source of weight stigma towards pregnant women and can shape stigmatisation across other socioecological levels. For instance, family, friends, colleagues, healthcare providers, and media professionals are all members of the broader community and tend to be influenced by dominant societal norms and discourses [70], where pervasive weight stigma reinforces cultural attitudes and, in turn, individual behaviours [71]. Hailu et al. examined explicit and implicit weight bias and profiles of characteristics associated with harbouring weight bias against perinatal women across six countries (Australia, Canada, US, UK, Malaysia, and India) [16]. Explicit and implicit weight bias and stigmatising attitudes were harboured by participants across all included nations, although less pronounced in non-Western countries. Latent profile analysis highlighted a distinct relationship between controllability beliefs (believing that an individual can control their weight) and weight bias.

The mainstream and social media also play a powerful role in shaping societal attitudes and beliefs that reinforce weight stigma and promote harmful body ideals [72, 73]. Pregnant and postpartum women frequently experience weight stigma perpetuated by the media [74] typically through pressure to attain an idealised appearance during pregnancy and rapidly ‘bounce back’ to a pre-pregnancy body after childbirth. Media narratives regularly applaud celebrities who meet these expectations, reinforcing unrealistic standards [74]. At the same time, the lack of or negative portrayals and blaming of larger-bodied women further contribute to stigma and exclusion [75]. These messages are linked to negative self-perceptions, body image concerns, and weight bias internalisation [72, 73].

Weight stigma is also widespread within women’s close social networks, including their families of origin, acquired and extended families, and friends [4, 10, 32, 76]. A recent study in the US and Canada found that 86% of pregnant women reported experiencing weight stigma from individuals within their close social circles [32]. The weight stigma took the form of assumptions about their own or their baby’s health or their “lifestyle” choices based on weight gain; comparisons to idealised pregnant bodies; and remarks that caused them to feel inadequate as expectant mothers [77]. These experiences were significantly associated with adverse health outcomes, including chronic pain and symptoms of depression and anxiety [32].

### Policy

When broadly defined, policies include “frameworks, strategies, action plans, policies, strategic plans, recommendations, or public health and wellbeing plans that propose a course or principle of action in the context of government, schools, healthcare, workplaces, or community settings” [78]. Weight stigmatising community narratives drive the development of stigmatising policies, especially by perpetuating the narrative of individual responsibility for body size [78]. Even when acknowledging the complex mechanisms and environmental drivers of obesity, policies often implicitly perpetrate weight stigma through poor language choice and conflicting discourse such as assuming higher body weight always equates to poor health, sustained weight loss is widely achievable, and weight stigma motivates weight loss [79]. Policies that overtly or inadvertently perpetuate weight stigma also shape weight stigmatising clinical practice [15]. A 2025 mapping review explored obesity-related polices relevant to preconception, pregnant, and postpartum women and found that weight stigma is rarely or inadequately recognised [15]. Persistent themes of personal responsibility and maternal blame dominated, linking mothers’ larger body size with babies’ wellbeing. The review recommended more comprehensive recognition of weight stigma, involvement of lived experience voices of larger-bodied women in policy development, mobilisation strategies targeting weight stigma, and consistent use of non-stigmatising language and narratives in perinatal policy.

### Maternity Care

Policies are a key driver of weight stigma in maternity care, alongside structural factors and explicit and implicit biases [80]. Structural factors include institutional policies, cultural norms, and societal conditions that facilitate weight stigma and lie beyond individual control. In healthcare, structural factors reinforce weight stigmatising experiences by influencing individual behaviour [81, 82]. Specifically, structural weight stigma manifests through institutional practices, professional norms, and societal conditions that condone stigma [80]. A systematic review explicated structural factors in reproductive healthcare, showing that in maternity care, this results in increased monitoring of larger-bodied women, often through the medicalisation of their care when focusing on the consequences of higher BMI [80]. Paradoxically, larger-bodied pregnant women also received less support and experienced poorer patient-provider relationships [80].

Recent work has highlighted midwives’ reflections of how healthcare professionals have the potential to negatively impact women’s care [2]. An integrative review illustrated how interpersonal, societal, organisational, and system-level factors influence midwives’ attitudes and beliefs, many of which are biased towards larger-bodied women [2]. Some midwives held stigmatising attitudes, reinforced by a perceived need for further training in caring for larger-bodied women and communicating the associated risks [2, 83]. Obstetricians/gynaecologists have been reported to hold more negative attitudes towards pregnant patients with higher BMIs than family physicians [13]. Positively, more recent research suggests that medical students may exhibit lower explicit biases towards pregnant patients than a decade ago and that maternity care providers report high levels of empathy attributed to broader societal trends towards body positivity [84, 85]. The Body Positivity movement, rooted in fat-acceptance and feminism, challenges dominant beauty ideals by promoting body acceptance, appreciation, and self-empowerment [86–88]. Collectively, this research highlights that reducing weight bias among clinicians remains necessary despite recent improvements.

### Gestational Diabetes Mellitus (GDM) Stigma

Stigma related to GDM is an emerging, increasingly recognised field of research intersecting with weight stigma in pregnancy [89]. Davidsen et al. identified GDM stigma as a global issue manifesting as both experienced and internalised stigma, with negative implications for psychological wellbeing and health behaviours among women with GDM. Subsequent qualitative studies in Denmark, the UK, the US, and Australia have documented women’s experiences of GDM stigma from healthcare personnel or family and friends, structural blame narratives, and self-blame [90–94]. Larger-bodied women reported being asked to repeat GDM testing when results contradicted clinician expectations [49]. These findings suggest GDM stigma is shaped by both interpersonal interactions and healthcare structures, often echoing or compounding weight-related stigma. Internalised GDM stigma, manifesting as guilt and self-blame, has been observed across multiple studies, particularly among women with prior overweight or a family history of type 2 diabetes [90–92]. Quantitative evidence indicates that self-blame often persists beyond pregnancy, with one in six women reporting self-blame for developing GDM one year after birth and one in three reporting self-blame related to potential adverse outcomes in their children [95]. Similar to weight stigma, GDM stigma has been associated with avoidance of healthcare, non-disclosure of the diagnosis, loneliness, disordered eating, and reluctance to attend follow-up care [89, 90], significantly impacting maternal wellbeing.

## Solutions Addressing Weight Stigma in Pregnancy

There have been no efficacy or effectiveness interventions addressing or targeting weight stigma in pregnancy across any of the socioecological levels. However, in non-pregnant populations, Talumaa et al. identified five stigma reduction strategies in healthcare [96]. These were increased education about weight stigma and its harms; education on obesity’s complex causes and controllability; evoking clinician empathy; providing weight-inclusive care; and approaches combining these strategies. The authors called for weight stigma to be addressed early in clinician training and continuously throughout healthcare education. Similar calls emphasise clear, non-stigmatising, pragmatic training that recognises biases and leverages the “do no harm” tenet of clinical practice [97]. Clinicians should use open, sensitive, patient-centred communication [98].

One approach addressing weight stigma that aligns with the “do no harm” principle of medical ethics and core tenets of midwifery care, including respect, safety, and woman-centred care, is weight-inclusive care [99–101]. A weight-inclusive approach prioritises non-weight-related health processes and outcomes to promote health and wellbeing [101]. It questions the assumption that a specific BMI reflects certain health behaviours, overall health status, or moral character. By doing so it helps to minimise stigmatisation in healthcare and improves physical, psychological, and behavioural health outcomes [102, 103]. Outside pregnancy, weight-inclusive interventions have demonstrated positive impacts on blood pressure, health behaviours, binge eating, depressive symptoms, and self-esteem [102, 104]. Women with lived experience of weight stigma in pregnancy described how weight-inclusive approaches to their care reinforced perceptions of provider commitment to the women’s autonomy and informed decision-making [3].

Researchers have also sought women’s perspectives on how to reduce weight stigma in perinatal settings [18, 20, 105]. Recommendations focused on clinical interactions acknowledging the complexity of obesity; considering individual circumstances and weight history; addressing weight in a positive and supportive manner; acknowledging underlying factors associated with body size; treating women as individuals not statistics; and reducing emphasis on weight [20]. Hill et al. co-designed antenatal care resources with midwives and women who had lived experience of weight stigma during their maternity care [18]. The resources addressed women’s need for representation through images exemplifying diverse bodies, countering high-risk assumptions in pregnancy related to body size, and promoting clinician empathy and education. Participants also highlighted stigmatising hospital policies as a significant priority to address weight stigma [18].

Interventions addressing weight stigma, including weight-inclusive care, should be supported with structural changes to prevent environments and policies from reinforcing weight stigma [80, 97], including pragmatic examples of reducing weight stigma in practice [18]. Further research should also explore the effectiveness of weight stigma interventions during pregnancy, including the impact on women’s psychosocial wellbeing and pregnancy outcomes.

## Conclusion and Future Directions

Weight stigma in pregnancy and related contexts is associated with adverse maternal and infant health impacts across socio-emotional, behavioural, and physical domains. Substantial evidence highlights these impacts and their role in perpetuating inequities from both lived experience and empirical research. Drivers of weight stigma in pregnancy manifest across socioecological environments, reinforcing stigma in policy and practice and perpetuating stigmatising narratives that blame women for larger body size and associated adverse outcomes. Consequently, to tackle weight stigma, evidence supports shifting from weight-centric maternity care to weight-inclusive, health-focused care centred on women’s needs.

Yet, substantial research gaps remain in efforts to end weight stigma in pregnancy and the broader perinatal period. First, greater involvement of people with lived experience across the research spectrum is needed to centre women’s voices. Secondly, research should address cultural and intersectional factors that contribute to weight stigma to better inform tailored solutions and reduce health inequities. Further to socioecological approaches that address community, media, and interpersonal weight stigma, strategies within healthcare are crucial. Hence, exploration of relationships between weight stigma and adverse pregnancy and birth outcomes, and causal mechanisms in associations between pre-pregnancy BMI, GWG, and adverse pregnancy and birth outcomes, should present findings in absolute risk to avoid overestimation. Such evidence could help dismantle stigmatising narratives and formulate a strong evidence-base for weight-inclusive care. Alongside evidence to inform less-stigmatising policies and structural environments pertinent to pregnant women and maternity care, clinicians also need training and solutions to reduce weight stigma in practice. Finally, evidence-based interventions addressing weight stigma in pregnancy, including weight-inclusive approaches, need to be developed and evaluated to provide a roadmap for the future. Ultimately, ending weight stigma in pregnancy has the potential to improve pregnancy and birth outcomes, with intergenerational benefits for the health and wellbeing of women and their families.

## Key References

- Davidsen E, Maindal HT, Rod MH, Olesen K, Byrne M, Damm P, Nielsen KK. The stigma associated with gestational diabetes mellitus: A scoping review. EClinicalMedicine. 2022 Aug 11;52:101614. https://doi.org/10.1016/j.eclinm.2022.101614
  - ○ This review of current evidence of the experiences of women with gestational diabetes mellitus (GDM) highlighted reporting of experienced and internalised stigma by women with GDM. Consequences of this stigma included avoidance of screening, not following dietary recommendations nor reporting blood glucose readings, social isolation, and poor mental wellbeing. 
- Dodd, J. M., Deussen, A. R., Poprzeczny, A. J., Slade, L. J., Mitchell, M., & Louise, J. (2024). Investigating discrepancies in findings between rigorous randomized trials and meta-analyses evaluating pregnancy interventions to limit gestational weight gain. Obesity Reviews, 25(12), e13826. https://doi.org/10.1111/obr.13826
  - ○ This study investigated the effect of trial quality on treatment effect estimates for interventions designed to limit gestational weight gain, challenging existing reports by highlighting that interventions had a clinically insignificant intervention effect of only 1.1 kg with no impact on any clinical outcomes. Hence, attempting to influence gestational weight gain has limited clinical benefit.
- Griffiths, A., Kingsley, S., Mason, Z., Tome, R., Tomkinson, M., & Jenkinson, B. (2023). Listening to larger bodied women: Time for a new approach to maternity care. Women and Birth, 36(5), 397-400. https://doi.org/10.1016/j.wombi.2023.07.001
  - ○ This paper centralises the lived experience voice as essential evidence to understand women’s experiences of weight stigma in maternity care. The lived experience authors call upon researchers and maternity care providers to recognise the unintended harms of weight-centric, stigmatising health policies, systems, and practices. The women simply ask for an approach to maternity care that prioritises being supported and feeling heard.
- Hailu, H., Skouteris, H., Incollingo Rodriguez, A. C., & Hill, B. (2024). SWIPE: A conceptual, multi-perspective model for understanding and informing interventions for weight stigma in preconception, pregnancy, and postpartum. Health Psychol Rev, 1-17. https://doi.org/10.1080/17437199.2024.2333801
  - ○ This paper presents a comprehensive, intervention-guiding conceptual model aimed at eliminating weight stigma for perinatal women. It recognises the multiple socioecological contexts where weight stigma occurs while identifying key touch points for intervention across the processes, psychological aspects, and social factors underlying weight-based stigmatisation along with the predictors and moderators of weight stigma and its formation.
- Schwenk, R. A., Wyss, C., & Aubry, E. M. (2025). Experiencing weight stigma during childbirth increases the odds of cesarean birth. BMC Pregnancy and Childbirth, 25(1), 191. https://doi.org/10.1186/s12884-025-07251-6
  - ○ The first study to explore the association between weight stigma and pregnancy outcomes. It identified that higher frequency of weight stigma experiences partially mediated the relationship between higher pre-pregnancy BMI and cesarean birth, demonstrating the potential for weight stigma experiences to be on the causal pathway to adverse pregnancy outcomes.

## Data Availability

No datasets were generated or analysed during the current study.

## Footnotes

1 The majority of weight stigma research in the reproductive period has been conducted with people identifying as women. Therefore, we use the term woman and not pregnant person in this paper. We do not intend to exclude research with diverse populations and acknowledge the varied weight stigma experiences of the LGBTIQA+ community.
